# Editorial: Temporal structure of neural processes coupling sensory, motor and cognitive functions of the brain, volume II

**DOI:** 10.3389/fncom.2023.1291744

**Published:** 2023-09-27

**Authors:** Daya Shankar Gupta, Arpan Banerjee, Federica Piras, Dipanjan Roy

**Affiliations:** ^1^School of Pharmacy, South University, Savannah, GA, United States; ^2^Cognitive Brain Dynamics Lab, National Brain Research Center, Gurugram, India; ^3^Laboratory of Neuropsychiatry, Department of Clinical Neuroscience and Neurorehabilitation, IRCCS Santa Lucia Foundation, Rome, Italy; ^4^Centre for Brain Science and Applications, School of Artificial Intelligence and Data Science, Indian Institute of Technology, Jodhpur, India

**Keywords:** consciousness, perception, binding, voluntary control, temporal, motor noise, active inference, embodied embedded cognition

Information processing is inextricably linked to the multi-scale temporal structure of the brain and the physical surroundings. The contributions to the Volume II of Frontiers Research Topic, “*Temporal Structure of Neural Processes Coupling Sensory, Motor and Cognitive Functions of the Brain*,” brings us a step closer to understanding how the coupling between the temporal structures of neuronal spiking activities and external events would contribute to voluntary motor behavior and perceptual functions. Shannon and Weaver (1949) argued that an information source generates a message, which is transmitted to a recipient. In the case of the brain, the activities of the cortex resulting from the sensory inputs and motor activities constitute the information source. Sensory inputs and motor actions, stemming from the interaction of the brain with external stimuli, encode messages in the brain's activity patterns that are decoded by the temporal coupling of neuronal spikes of the brain [i.e., the association between neural activities, reflecting increased mutual information (Gupta and Bahmer, 2021)] due to the demands of a task (Gupta and Bahmer, 2019). Temporal coupling reduces average uncertainty in the message, which results in the “knowledge,” underlying voluntary motor control and perception.

The message/information from the sources in the brain, such as the concerted activities of sensory and motor areas during a task execution is assessed by the measurement of average uncertainty or Shannon entropy, hereafter called entropy. As the amount of entropy (the message/information that has to be decoded) gets bigger, then the voluntary control exerted by the individual improves, as well as there is an increase of the likelihood of veridical outcomes derived from sensory tasks. Consistent with the preceding, Wang et al. reported that there is an increase in motor variability whenever the subjects received negative feedback regarding performance in a motor timing task. In this task, the subjects made delayed saccadic eye movements or button presses in response to a cue. An increase in motor variability implies an increase in the average uncertainty or entropy, which means that there is an increase in the amount of processed information for achieving better control of the timing task in the next trial (i.e., heightened voluntary regulation of movements).

Wang et al. proposed that feedback is used to tune the motor variability in humans. To study their hypothesis, they compared different recurrent neural network (RNN) models, which included graded recurrent units (GRU), a vanilla autoregressive neural network (ARNN), and weight-varying ARNN (vARNN) to analyze which one best captures experimental human data. vARNN stood out as the best network model in terms of its prediction accuracy based on the history (previous trial). In vARNN architecture, an adjustable noise level was added to the connectivity between units, which was modulated, on a short-term basis, by feedback. The authors contend that feedback may be responsible for modulating the motor variability in humans, that is, “reducing the variability when the outcome is better than expected (less exploration) and increasing the variability when the outcome becomes worse.”

Boenke et al. studied pre-stimulus alpha and beta wave modulation in a self-paced, cue-independent, spatialized temporal order judgment (TOJ) task in auditory and visual modalities. Boenke et al. reported “on the group level: (1) Veridical auditory TOJs, relative to non-veridical, were associated with higher (pre-stimulus) 20 Hz (beta) power over central electrodes, and (2) veridical visual TOJs showed higher (pre-stimulus) 10, 15 Hz (alpha beta) power over parieto-occipital electrodes.” Moreover, in this study, “the individual-level modulation pattern was variable and included activations opposite to the group mean.” The authors also observed that the direction of individual activation of electrodes over auditory brain regions and parietooccipital electrodes was always negatively correlated in the respective TOJ conditions. Based on this, the authors argue against dismissing individual variations as outliers. Instead, they argue that there are different strategies (reflected in opposite directions of modulations of brain oscillations) used by the brain leading to the same outcome, namely veridical TOJs in their study. Thus, as advised by the authors, a general description of brain activity during the processing of incoming information should account for variability in modulation directions at both the group and individual levels.

Also note that positive modulation, which is synchronization, will concentrate spiking activities to one particular phase of the oscillation cycle, reducing the entropy; and negative modulation, which is desynchronization, will increase entropy in spiking patterns. Furthermore, desynchronization frees up neurons from the restriction of spiking only during a certain phase of oscillations. Therefore, desynchronization will promote the temporal coupling between neuronal activities, given the constraints of interactions involving external stimuli. In this context, it is also noteworthy that a decrease in the synchronization of beta oscillations across sensorimotor areas is seen during movement execution (Barone and Rossiter, 2021), which will immensely increase entropy during the post-stimulus phase.

Based on the preceding discussion, the findings by Boenke et al. may also be interpreted in the light of information-theoretic considerations of cognitive functions (Gupta and Bahmer, 2019), as one can argue that lower entropy measured at the beginning of the task (the observed high pre-stimulus modulation) would lead to a greater quantitative increase in entropy, which will increase the chances of a veridical TOJ. In this context note that a greater increase in entropy would allow greater levels of reduction of average uncertainty (entropy) in the brain activities via temporal coupling, resulting from the specific constraints for successful task completion. Since, at the group level, there is an increase in alpha/beta modulation in the pre-stimulus stage, consequently lower initial entropy, it will lead to a greater increase in entropy as the task is completed, contributing to its success. However, at an individual level, there may be a negative modulation in the respective modality, which is, however, accompanied by positive modulation in the non-attended modality. In those individuals, there still could be a net decline in entropy in the pre-stimulus stage due to positive modulation of brain oscillations in non-attended modality areas, contributing to an overall increase in the amount of entropy as the task is completed. However, it is not clear how a quantitative increase in information (entropy) is responsible for veridical TOJs. A net increase in entropy could be related to an enhanced state of alertness, resulting from the ability for a greater decrease in uncertainty via temporal coupling.

Liu et al. used a biologically constrained, large-scale neural network model (LSNM) of short-term memory to study how endogenous (top-down) and exogenous (bottom-up) attention are controlled in an intersensory auditory- and visual-perception paradigm. The LSNM consists of several interconnected modules, which were embedded into the structural connectome of Hagmann et al. (2008). The modules are made of arrays of basic units, each consisting of one excitatory subunit and an inhibitory subunit, mimicking cortical minicolumns. The authors have used simulated subjects, created by slightly varying interregional structural connection weights through a Gaussian process. Two types of tasks are used: delayed match-to-sample and Sternberg tasks. Whenever there is a match of the probe stimulus with previously presented stimuli during a task, the response submodule issues a response. The visual or auditory stimuli are sent to a buffer simulating working memory processes by increasing endogenous attention from low (0.1) to high (0.3). The exogenous attention to stimuli, including distractors, is augmented by increasing stimulus saliency. The results show that increasing the working memory load in one modality would reduce distraction from the other modality. Simulated fMRI data were also generated. The authors hope that these predictions can be tested on human subjects although they argue that computational modeling can be a powerful tool for interpreting and integrating neuroimaging data coming from different sources (human and non-human).

Löffler and Gupta presented a computational model, using three ensembles of neurons, for reduction in spatiotemporal overlap of spiking patterns resulting from different inputs. Three ensembles include input (I), R (temporal separation), and E (temporal separation to spatial separation). I neurons are randomly connected to R neurons. In this model, a traveling Excitatory Post-Synaptic Potential (EPSP) wave activates a spatially different E neuron when the peak of the traveling EPSP wave coincides with output from R neurons. This leads to a perfect conversion of temporal separation patterns into spatial patterns. Thus, a temporally separate output will activate a different neuron/or minicolumn in a contiguous area of the cortex. Activations of separate minicolumns or cortical neurons can be independently temporally coupled to each other, based on the constraints resulting from a task, argued to play a role in voluntary motor control and perception (Gupta and Bahmer, 2019). The authors also studied object identification. They used three inputs into eight I (input) neurons, representing three colors, three shapes, and the two sizes of an object (18 different objects). The spatiotemporal separation, representing three groups of features, was about 90%. Since spatiotemporal separation does not exceed 100% in the above example, this mechanism may not lead to an increase in entropy, given the completion of a task. Nonetheless, this model provides a mechanistic basis for the spatial separation of input patterns that would play a role in object identification.

The contributions in this Research Topic shed light on how the temporal structure, represented at multiscale levels, may affect information processing underlying cognitive functions of the brain. Wang et al. show that negative feedback immediately increases motor variability, which may be a response to reduce errors in a motor timing task. A quantitative increase in entropy, a measurement of the amount of information, appears to be a common feature in disparate strategies to achieve veridical TOJs in the study by Boenke et al.. Papers by Liu et al. and Löffler and Gupta show how different computational approaches can be useful in understanding the complex dynamics of information processing underlying cognitive functions.

## Author contributions

DG: Writing—original draft. AB: Writing—review and editing. FP: Writing—review and editing. DR: Writing—review and editing.
